# Supplementary material for: Mutations in SLC45A2 lead to loss of melanin in parrot feathers
Source: G3 (Bethesda). 2023 Nov 7;14(2):jkad254. doi: 10.1093/g3journal/jkad254 (PMC10849330; doi:10.1093/g3journal/jkad254)
Supplement: jkad254_Supplementary_Data [file jkad254_supplementary_data.zip › Supplemental_Material_Legends_G3-2023-404649.docx]

**Figure S1:** Non-synonymous protein-coding variations found in SLC45A2 homologs. A) Missense mutations G467V, G399R and P53L found in rose-ringed (*P. krameri*). B) Missense mutations L180P and G399R found in alexandrine parakeet (*P. eupatria*). C) Nonsense mutation R25* found in Plum-headed parakeet (*P. cyanocephala*). D) Positions of nucleotide changes at different exons of *SLC45A2* homologs.
