# Supplementary material for: Mutations in SLC45A2 lead to loss of melanin in parrot feathers
Source: G3 (Bethesda). 2023 Nov 7;14(2):jkad254. doi: 10.1093/g3journal/jkad254 (PMC10849330; doi:10.1093/g3journal/jkad254)
Supplement: jkad254_Supplementary_Data [file jkad254_supplementary_data.zip › Supplemental_Tables_S1-S3_G3-2023-404649.pdf]

Supplementary tables

Table S1: Intronic primers used for *P. krameria, P. eupatria* and *P. cyanocephala.*

Primer name	Sequence
EX1 F	GCCAGCACGCCGCACATAGC
EX1 R	ATTCAAAAGGCTAGGATGAG
EX2 F	GCAGAACATGGTACTGTTGC
EX2 R	GATAAAATCCATGCAGTTAC
EX3 F	TTTATCAGTGCCAGTAGACC
EX3 R	TTAGATTAAAGGGTTCCAGG
EX4 F	AAGATATAAAGGCTGTCTCC
EX4 R	ATGCTAAGAAGAATGAGAAG
EX5 F	GAGGCCTTGTGTTGGCCACC
EX5 R	AAAGCACAGAGTTGGAGTGC
EX6 F	TACCTACACTAATTTGAGGC
EX6 R	GACTAAACAGACCCAGACGC
EX7 F	TGCAAGTTGTCAGCACCGTC
EX7 R	CTTCCTTCCAATCACTTCAC

Table S2: Primers used for cloning *PkSLC45A2*.

Primer name	Sequence
PkSLC45A2_F	AGAATAAACGCTCAACTTTGGGCCCCTCGAGGTCGACATGGACAGCACCAAAGAAGAG
PkSLC45A2_R	TGGTAACCAGATCTCCCGGGCCGCGGAATTCGTCGACCTACTCCACATAGTGTACAC
HA_SLC45A2 F	ATGTATCCGTATGATGTTCCGGATTATGCAGGCAGCGACAGCACCAAAGAAGAGGAG
SLC45A2_KpnI R	CAATCTTTCACAAATTTTGTAATCCAGAGGTTGATTAGGATCTATCGATTCAGGTACCCT ACTCCACATAGTGTACAC
pHAGE_KpnI_ HA F	GCCTGAAATCACTTTTTTTCAGGTTGGACCGGTGCCACCCATCTCGAGGGTACCATGTAT CCGTATGATGTTCC

Table S3: Primers used for introducing mutations into *PkSLC45A2* coding sequence.

**Primer name**	**Sequence**
C188T_SLC45A2_F	GCCGTGGAGGCCGCCTTTGTCACGCtGGTGCTGCTCAGTGTAGGGCTGCC
C188T_SLC45A2_R	GGCAGCCCTACACTGAGCAGCACCaGCGTGACAAAGGCGGCCTCCACGGC
T569C_SLC45A2_F	GAAAGGTCTGCATTACCACGCCCcCCTCACAGGTTTGGGAGGAGCCCTGGG
T569C _SLC45A2_R	CCCAGGGCTCCTCCCAAACCTGTGAGGgGGGCGTGGTAATGCAGACCTTTC
G1225A_SLC45A2_F	GGACTAAAGGGACTTTATTTCATCaGATACCTACTTTTTGGTTTGGGTAC
G1225A_SLC45A2_R	GTACCCAAACCAAAAAGTAGGTATCtGATGAAATAAAGTCCCTTTAGTCC
G1430T_SLC45A2_F	CTGGACAGCCTGGGCGAGGGAAGGtCATTGACTGTGCTGCTCTCACCTCC
G1430T_SLC45A2_R	GGAGGTGAGAGCAGCACAGTCAATGaCCTTCCCTCGCCCAGGCTGTCCAG
