# Supplementary figures and images for: Mutations in SLC45A2 lead to loss of melanin in parrot feathers
Source: G3 (Bethesda). 2023 Nov 7;14(2):jkad254. doi: 10.1093/g3journal/jkad254 (PMC10849330; doi:10.1093/g3journal/jkad254)

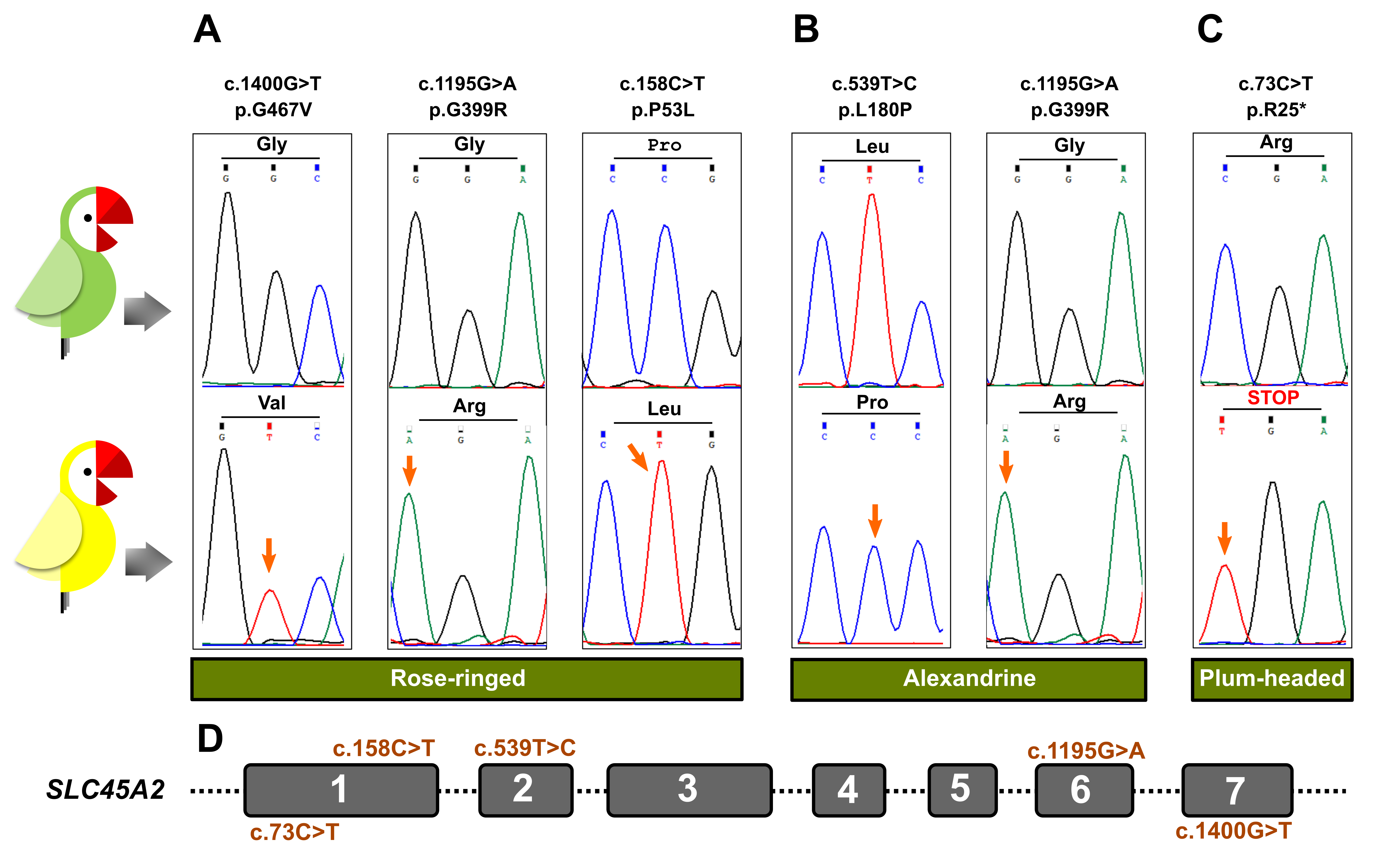

Supplement: jkad254_Supplementary_Data [file jkad254_supplementary_data.zip › 07.12.23_S1.png]
